# Salvage surgery for stage IVa thymic carcinoma combined with aortic arch resection – case report

**DOI:** 10.1186/s13019-020-01354-1

**Published:** 2020-10-07

**Authors:** Hiroyuki Yamato, Soichiro Funaki, Kazuo Shimamura, Keiwa Kin, Toru Kuratani, Yoshiki Sawa, Yasushi Shintani

**Affiliations:** 1 Department of General Thoracic Surgery, Osaka University Graduate School of Medicine, Osaka, Japan; 2 Department of Cardiovascular Surgery, Osaka University Graduate School of Medicine, Osaka, Japan

**Keywords:** Thymic carcinoma, Salvage surgery, Aortic arch replacement, Pneumonectomy

## Abstract

**Background:**

Although complete surgical resection of thymic carcinoma is a prognostic factor, extended surgery combined with a major blood vessel procedure remains controversial because of the increased risk of mortality. We report a case of Stage IVa thymic carcinoma successfully resected with a pneumonectomy along with aortic arch replacement after chemotherapy.

**Case presentation:**

A 45-year-old male was diagnosed with thymic carcinoma invasion to the aortic arch and left pulmonary artery. Malignant pericardial effusion was also noted, though disappeared after chemotherapy, thus surgical options were considered. A radical resection procedure including left pneumonectomy, aortic arch replacement with total rerouting of the supra-arch vessels, and right pulmonary artery plication was performed. The postoperative course was uneventful and the patient has been disease-free for 3 years.

**Conclusion:**

Extended salvage surgery might be a valuable option for advanced thymic carcinoma.

## Background

A thymic carcinoma is typically asymptomatic, thus invasion of adjacent structures is usually first noted at the time of diagnosis [1]. Since survival is significantly better for patients who undergo a complete resection, it is necessary to perform an extended operation [2]. We report here successful resection of a Stage IVa thymic carcinoma performed using salvage surgery combined with a pneumonectomy and great vessel replacement after chemotherapy.

## Case presentation

**Fig. 1:**
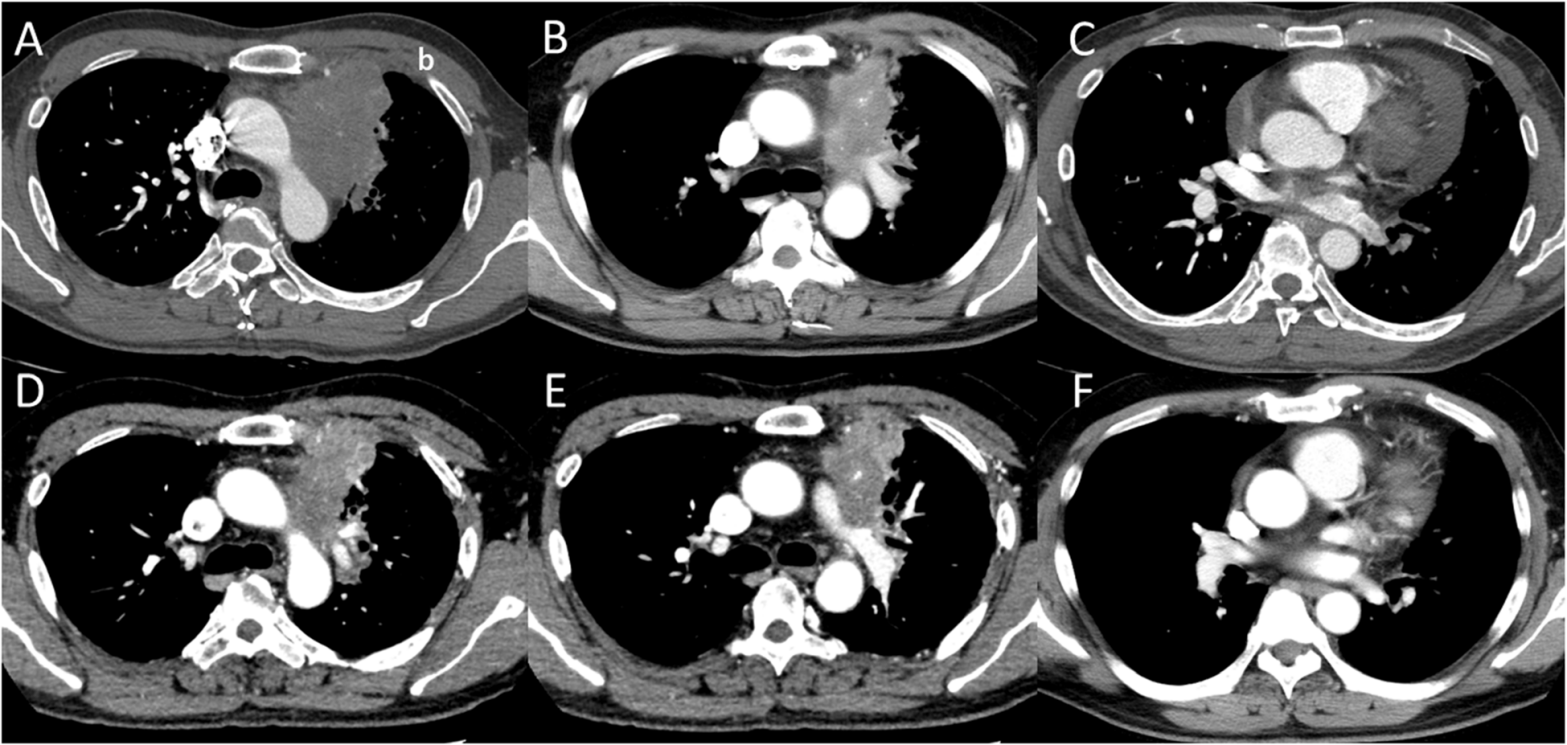
Radiographic images obtained (**a**-**c**) before and (**d**-**e**) after chemotherapy. Chest computed tomography (CT) findings showed an anterior mediastinal tumor invading the (**a**) aortic arch and (**b**) left hilar part of the left lung, as well as (**c**) pericardial effusion. After the end of the course of chemotherapy, CT showed that the tumor had shrunk, though invasion to (**d**) the aorta and (**e**) left pulmonary artery was still evident, while (**f**) pericardial effusion had disappeared. Lt PA, left pulmonary artery

**Fig. 2:**
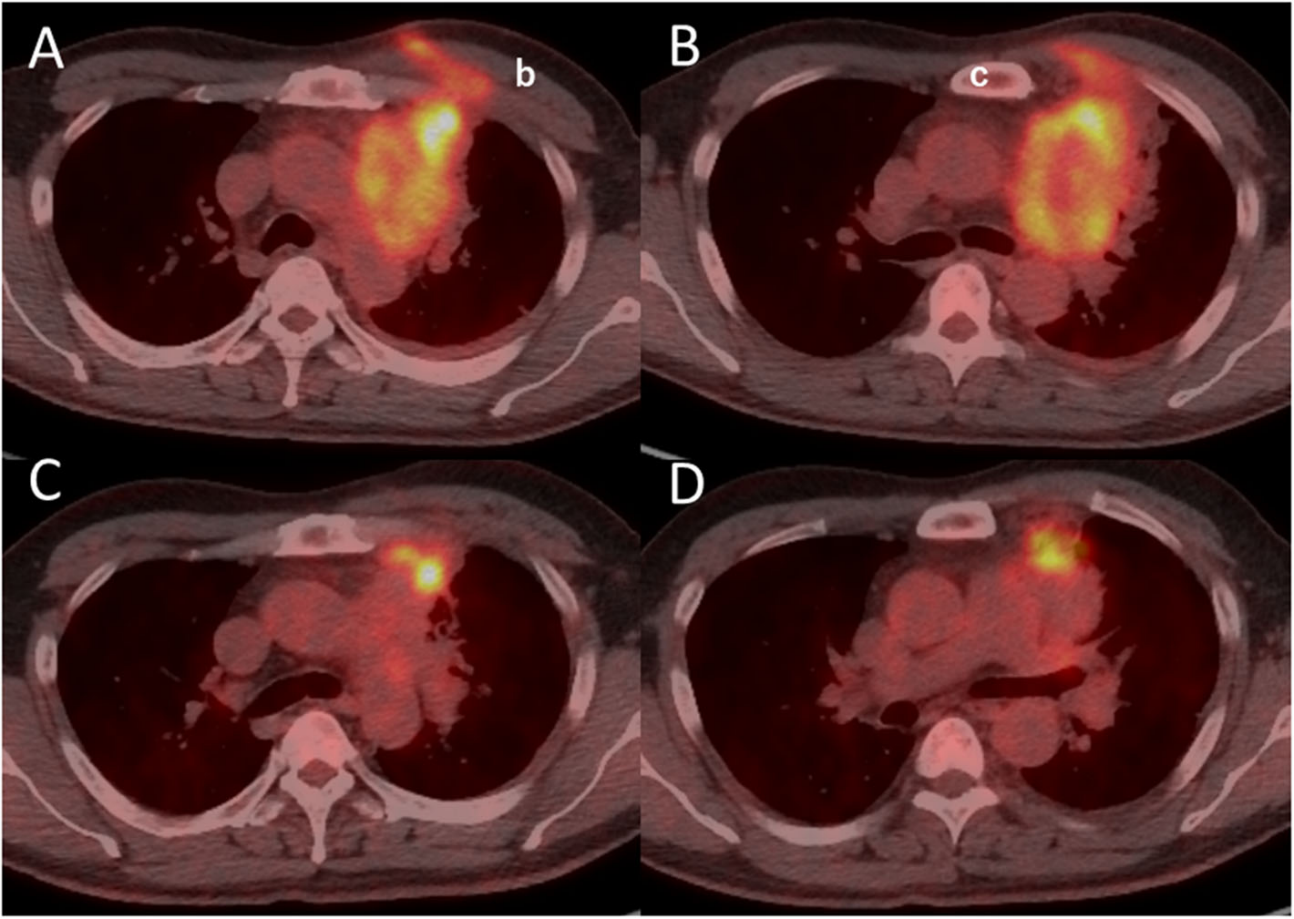
Fluorodeoxyglucose-positron emission tomography images obtained (**a**-**b**) before and (**c**-**d**) after chemotherapy

A 45-year-old male was diagnosed with an anterior mediastinal tumor and referred to our hospital. Open biopsy results of the tumor revealed a squamous cell carcinoma and cytology findings were positive for pericardial effusion. Chest computed tomography (CT) showed a mass approximately 10 cm in size with invasion to the left hilar part of the left lung and aortic arch, as well as pericardial effusion (Fig. 1a-c), thus the patient was diagnosed with a thymic carcinoma, c-Stage IVa (cT4N0M1a). Six courses of chemotherapy with carboplatin and paclitaxel were performed, followed by tegafur/gimeracil/oteracil (S-1) administration for 1 year. Chest CT findings showed disappearance of pericardial effusion and slightly decreased tumor size (Fig. 1d-f), while fluorodeoxyglucose (FDG)-position emission tomography also revealed that FDG uptake was decreased after the chemotherapy regimen (Fig. 2). However, at this time the patient was affected by liver dysfunction due to chemotherapy, thus our multidisciplinary team considered surgical options because that administration could not be continued.

**Fig. 3:**
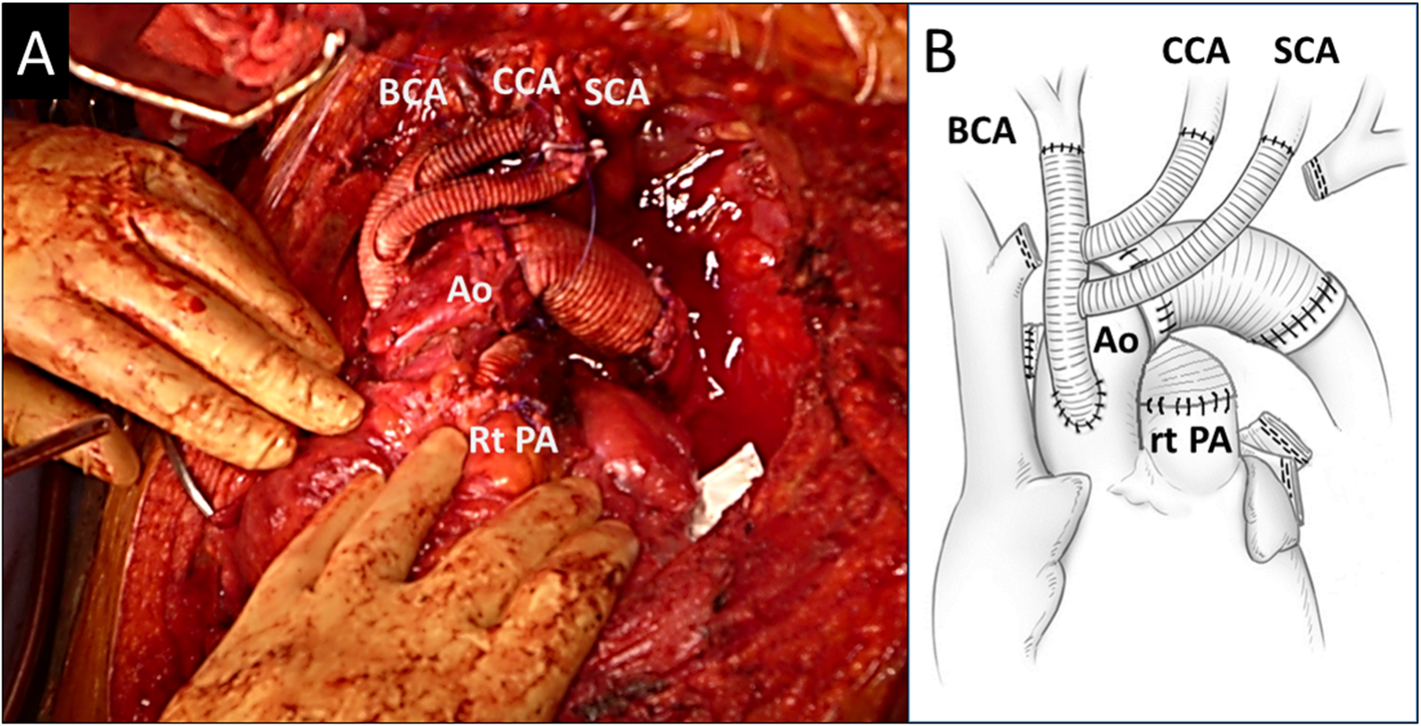
**a** Intraoperative image showing aortic arch replacement using total rerouting of supra-arch vessels combined with right pulmonary artery replacement. **b** Schematic drawing of surgical procedure. Ao, aorta; BCA, brachiocephalic artery; CCA, common carotid artery; Rt PA, right pulmonary artery; SCA, subclavian artery

Preoperative systemic restaging was yc-Stage IIIb (yc-T4N0M0). A salvage operation including aortic arch replacement was considered to be challenging, thus we carefully explained the risk of surgery to the patient and his family, and obtained informed consent. A left lateral thoracotomy was initially performed, and the findings ruled out pleural or pericardial dissemination, thus a median sternotomy was added. Invasion of the chest wall by the tumor was noted, thus resection of the chest wall 8 × 5 in size as well as the pectoralis major muscle was performed. The tumor was suspected to have invaded the main pulmonary artery (PA) trunk as well as the aortic arch. The left brachiocephalic vein showed obvious tumor invasion and was dissected, though the mass could not be divided from the aorta. Moreover, the left PA could not be encircled in the pericardium. Following systemic heparinization (300 U/kg), a cardiopulmonary bypass (CPB) was established with right atrium drainage, as well as 2 points of arterial perfusion via the femoral and right axillary arteries. The shrunken left main trunk of the PA was then dissected and divided with a stapler, and the upper and lower pulmonary veins, and left main bronchus were also divided. Finally, the tumor was sharply separated from the aorta and removed along with the left lung. The left recurrent laryngeal and phrenic nerve were involved in the tumor and resected, whereas the cutting edge was negative for viable tumor cells in the pericardium and pulmonary artery, as shown by frozen section findings. Some tumor residue remained on the aortic wall and was confirmed to be viable, thus a residual tumor resection with replacement of the aortic arch using total rerouting of the supra-arch vessels [3] was performed. To confirm that no tumor remained other than in the aortic wall, selected points such as fat near the pulmonary artery and the aorta in the remaining area were confirmed to be negative by frozen section findings. Anastomoses of the ascending aorta and trunk of the trifurcation graft (Hemashield three-branch graft, 12–8-8 mm) were performed with side-clamping, and subsequently the brachiocephalic artery, left common carotid artery, and left subclavian artery were reconstructed one by one using a simple clamping method. Next, after clamping the ascending aorta just distal to the trifurcated graft inflow anastomosis and proximal descending aorta, the aortic arch was resected with the residual tumor and reconstructed using a 26-mm graft. Cardiac arrest was not introduced at any time during the procedure, though CPB could not be weaned because of right heart failure caused by PA bifurcation stenosis. Therefore, we reconstructed the PA bifurcation, the stenosis of which was due to the division line of the left PA being too close to the PA trunk, using an 18-mm tube graft (Fig. 3) for replacing the PA trunk and right PA. After PA repair, CPB was weaned uneventfully. For repair of the defect in the chest wall, a polypropylene mesh was fixed to the chest wall when the chest was closed. The operation time was 958 min and CPB time was 254 min, while blood loss was 7980 ml. The patient was extubated on postoperative day (POD) 2 and the postoperative course was uneventful. Pathological diagnosis results revealed that viable tumor cells were present in the resected aortic wall, indicating that the final pathological stage was IIIb (yp-T4N0M0). The patient has been disease-free for 3 years.

## Discussion and conclusions

Few cases of thymic carcinoma have been reported and consensus regarding treatment other than complete resection is lacking [4]. A patient with a locally advanced thymic carcinoma invading the heart or great vessels can be treated with radical surgical resection, though the risk of perioperative morbidity will increase. However, usage of CBP has been reported to improve the chance of complete tumor resection in selected patients and might lead to prolonged survival [5]. Furthermore, Petrella et al. noted that salvage surgery may represent the sole effective therapy for patients with thymic malignancy who do not respond to other curative treatments, and offers a chance for curative treatment in selected patients with acceptable morbidity and mortality [6].

The indications for salvage surgery with aortic resection should be carefully considered. We previously reported results of ascending aortic replacement [7] and aortic arch replacement [8] performed in a conventional manner for thymic carcinoma cases. In the present patient, though the tumor had shrunk and pericardial effusion disappeared after chemotherapy, the treatment could not be continued due to liver dysfunction, while irradiation therapy was not indicated because the lesion was wide and malignant pericardial effusion was present. Therefore, the only therapeutic treatment option considered to be relevant for this case was salvage surgery. Our novel technique for aortic replacement may be advantageous, because with it aortic arch resection can be completed under a tepid temperature and beating heart condition [3]. Furthermore, it has potential to avoid side-effects associated with deep hypothermic circulatory arrest and ischemia-reperfusion injury of multiple organs. Since the right PA was stenotic after resection, that was also replaced in the present case. The result was successful macroscopic complete resection performed as salvage surgery combined with replacement of the great vessels and the postoperative course was uneventful, even following aggressive surgery.

In conclusion, salvage extended surgery has been shown to improve the prognosis in select patients with advanced thymic cancer.

## Data Availability

Not applicable.

## Abbreviations

CPB: Cardiopulmonary bypass
CT: Computed tomography
FDG: Fluorodeoxyglucose
PA: Pulmonary artery
POD: Postoperative day
